# Effects of Dietary Crude Protein Levels and Cysteamine Supplementation on Protein Synthetic and Degradative Signaling in Skeletal Muscle of Finishing Pigs

**DOI:** 10.1371/journal.pone.0139393

**Published:** 2015-09-30

**Authors:** Ping Zhou, Lin Zhang, Jiaolong Li, Yiqiu Luo, Bolin Zhang, Shen Xing, Yuping Zhu, Hui Sun, Feng Gao, Guanghong Zhou

**Affiliations:** 1 College of Animal Science and Technology, Key Laboratory of Animal Origin Food Production and Safety Guarantee of Jiangsu Province, Synergetic Innovation Center of Food Safety and Nutrition, Nanjing Agricultural University, Nanjing, 210095, China; 2 College of Animal Science and Technology, Jilin Agricultural University, Changchun, 130118, China; National Institute of Agronomic Research, FRANCE

## Abstract

Dietary protein levels and cysteamine (CS) supplementation can affect growth performance and protein metabolism of pigs. However, the influence of dietary protein intake on the growth response of CS-treated pigs is unclear, and the mechanisms involved in protein metabolism remain unknown. Hence, we investigated the interactions between dietary protein levels and CS supplementation and the effects of dietary crude protein levels and CS supplementation on protein synthetic and degradative signaling in skeletal muscle of finishing pigs. One hundred twenty barrows (65.84 ± 0.61 kg) were allocated to a 2 × 2 factorial arrangement with five replicates of six pigs each. The primary variations were dietary crude protein (CP) levels (14% or 10%) and CS supplemental levels (0 or 700 mg/kg). The low-protein (LP) diets (10% CP) were supplemented with enough essential amino acids (EAA) to meet the NRC AA requirements of pigs and maintain the balanced supply of eight EAA including lysine, methionine, threonine, tryptophan, valine, phenylalanine, isoleucine, and leucine. After 41 days, 10 pigs per treatment were slaughtered. We found that LP diets supplemented with EAA resulted in decreased concentrations of plasma somatostatin (SS) (*P*<0.01) and plasma urea nitrogen (PUN) (*P*<0.001), while dietary protein levels did not affect other traits. However, CS supplementation increased the average daily gain (*P*<0.001) and lean percentage (*P*<0.05), and decreased the feed conversion ratio (*P*<0.05) and back fat (*P*<0.05). CS supplementation also increased the concentrations of plasma insulin-like growth factor 1 (IGF-1) (*P*<0.001), and reduced the concentrations of leptin, SS, and PUN (*P*<0.001). Increased mRNA abundance of Akt1 and IGF-1 signaling (*P*<0.001) and decreased mRNA abundance of Forkhead Box O (FOXO) 4 (*P*<0.01) and muscle atrophy F-box (*P<*0.001) were observed in pigs receiving CS. Additionally, CS supplementation increased the protein levels for the phosphorylated mammalian target of rapamycin (mTOR), eIF-4E binding protein 1, and ribosomal protein S6 kinase 1 (*P*<0.001). There were no interactions between dietary protein levels and CS supplementation for all traits. In conclusion, dietary protein levels and CS supplementation influenced growth and protein metabolism through independent mechanisms in pigs. In addition, LP diets supplemented with EAA did not affect growth performance and other traits except the concentrations of SS and PUN probably through maintenance of protein synthesis and degradation signaling. Moreover, CS supplementation improved growth performance by increasing plasma IGF-1 concentrations possibly through alterations of mTOR and Akt/FOXO signaling pathways in skeletal muscle of finishing pigs.

## Introduction

Cysteamine (CS; mercaptoethylamine, HS-CH_2_-CH_2_-NH_2_) is used as a feed additive in animal nutrition to improve the growth performance and feed efficiency of broilers, carp and pigs [1–3]. CS also reduces back fat thickness and improves the lean percentage of pigs [4]. Previous studies have demonstrated that CS decreases somatostatin (SS) levels in tissue and the hypothalamus, which leads to an increase in growth hormone (GH) secretion [3]. GH improves growth performance by increasing the production of insulin-like growth factor 1 (IGF-1), consequently stimulating protein synthesis and reducing the rate of protein degradation in muscle [5]. In addition, nitrogen from pig manure has contributed to the serious pollution of the environment [6]. Feeding low-protein (LP) diets supplemented with essential amino acids (EAA) is an effective strategy to reduce N excretion in swine and has no detrimental effect on growth performance of both growing and finishing pigs [7]. However, if dietary crude protein (CP) is reduced by more than 4 percentage units, it often leads to an unbalanced supply of amino acids (AA). This in turn, results in an impairment of pig performance [8]. It is well known that, only lysine, methionine, threonine, tryptophan and valine are commercially available in economic quantities. These AA can be added to LP diets to meet the NRC AA requirements of pigs. Some other EAA (e.g., phenylalanine, leucine, and isoleucine) also play an important role in keeping AA balance and maintaining growth performance [9,10]. However, there is little information about the influence of dietary protein intake on the growth response of CS-treated animals, and whether there is any interaction between dietary protein levels and CS supplementation remains unclear.

In adult animals, nearly all of the muscle mass is composed of skeletal muscle fibers [11]. The cell size of skeletal muscle is determined as a balance between protein synthesis and protein degradation [12]. The mammalian target of rapamycin (mTOR) plays a central role in cell growth and proliferation, and acts as a key regulator of protein synthesis [13]. Previous studies have revealed that Akt (protein kinase B, also known as PKB), an important upstream signaling protein of mTOR, activates mTOR, which in turn promotes protein synthesis through the phosphorylation and activation of ribosomal protein S6 kinase (S6K1), and the phosphorylation and inactivation of the mRNA translation repressor, known as eukaryotic initiation factor 4E-binding protein (4E-BP1) [14,15]. In addition, skeletal muscle atrophy occurs primarily through activation of the ubiquitin-proteasome pathway (UPP), which is the primary intracellular system for protein degradation in skeletal muscle [16,17]. Two genes encoding muscle-specific ubiquitin ligases, Muscle atrophy F-Box (MAFbx; also known as atrogin-1) and Muscle Ring finger 1 (MuRF1), are responsible for increased protein breakdown, and are rapidly upregulated in different models of muscle atrophy [18].

Some studies have demonstrated that IGF-1 and insulin not only induce skeletal muscle hypertrophy via the IGF-1 (insulin)/Akt/mTOR pathway but also reduce the expression of atrophy-related ubiquitin ligases, namely MAFbx and MuRF1, by inhibiting the forkhead box O (FOXO) family of transcription factors [19–21]. In a previous study, Liu et al., [22] reported that CS supplementation could regulate the gene expression of GH-IGF axis components (such as GHR, IGF-1, IGF-1R (IGF-1 receptor), etc.) *in vivo*. Additionally, the results of a protein turnover trial on finishing pigs showed that CS supplementation increased protein deposition due to a decrease in protein degradation, but it did not change the rate of protein synthesis [3]. However, the mechanisms responsible for the changes in anabolic and catabolic metabolism have not been delineated. Thus, the aim of the present study was to investigate how CS supplementation altered protein synthesis and proteolysis in the skeletal muscle of finishing pigs *in vivo*. Moreover, regulation of mTOR signaling is also induced by AA [23]. In skeletal muscle of neonates, feeding or acute infusion of AA could stimulate protein synthesis by increasing the phosphorylation of 4E-BP1 and S6K1 [24]. A deficiency and/or imbalance of EAA can decrease muscle protein synthesis [25]. Limited data suggested that a LP diet supplemented with deficient EAA (lysine, methionine, threonine, tryptophan, leucine, isoleucine and valine) suppressed protein synthesis partly by inhibiting mTOR signaling in weaned piglets. In addition, the phosphorylation levels of 4E-BP1 in skeletal muscle and mTOR in liver were shown to be decreased [26]. Nevertheless, whether a LP diet supplemented with enough EAA could ensure adequate protein synthesis in finishing pigs is unclear, and the mechanisms involved in protein metabolism in skeletal muscle remain unknown.

Therefore, the objectives of this study were to clarify whether there was a significant interaction between dietary protein intake and CS supplementation. In addition, we examined the effects of dietary protein levels and CS supplementation on the expression of genes associated with protein synthesis and degradation signaling pathways in the skeletal muscle of finishing pigs *in vivo*.

## Materials and Methods

### Animal Management and Dietary Treatments

One hundred twenty barrow (Yorkshire × Landrace × Meishan) pigs with an average initial body weight of 65.84 ± 0.61kg (mean ± SEM) were randomly allocated into four treatments using a 2 × 2 factorial arrangement with five replicates (pens) and six pigs each. The primary variations of this experiment were the dietary CP levels (14% or 10%) and CS supplemental levels (0 or 700 mg/kg). The commercial CS feed additive containing 30% of CS hydrochloride with starch and dextrin as carriers for stabilization was provided by Shanghai Walcom Bio-Chem Co. Ltd. (Shanghai, China). The pigs were fed with one of the four diets, as follows: [normal protein (NP) diet (14% CP), NP diet supplemented with CS, LP diet (10% CP), and LP diet supplemented with CS]. The NP diet was supplemented with lysine, methionine, threonine, and tryptophan. However, the LP diet was supplemented with enough EAA to meet the NRC AA requirements of pigs and maintain the balanced supply of eight EAA including lysine, methionine, threonine, tryptophan, valine, phenylalanine, isoleucine, and leucine (**Table 1**). The levels of AA supplementation were determined based on the standardized ileal digestible (SID) AA content (NRC, 2012) [27]. The concentrations of AA in the ingredients containing protein were analyzed and values for SID AA were estimated using SID coefficients provided by Xiong et al., [28] (**Table 1**). The SID content of leucine was 0.80% in the LP diets, which met the NRC leucine requirement of 75-100kg finishing pigs that was 0.74% [27]. Therefore, leucine was not supplemented to the LP diets. The ingredients and nutrient levels of basal diets at the two protein levels were formulated to meet the NRC (2012) [27] nutrient requirements of finishing pigs (**Table 1**) and to be isoenergetic with regard to the net energy (NE). The experiment was carried out at Jiangsu Academy of Agricultural Sciences (Nanjing, China). Feed and water were provided *ad libitum* throughout the whole experimental period. The duration of the experiment was 41 days. The experimental design and all the procedures were approved by the Animal Care and Use Committee of Nanjing Agricultural University.

**Table 1:** Ingredients and nutrient content of the basal diets.

Item	Diets	
	Normal protein	Low protein
**Ingredient (%)**		
**Maize**	75.43	76.50
**Soybean meal**	11.79	3.50
**Cottonseed meal**	4.00	0.70
**Wheat bran**	4.00	9.00
**Soybean oil**	1.60	0.15
**Maize starch**	—	6.0
**L-Lysine-HCL**	0.28	0.55
**DL-methionine**	0.02	0.11
**L-threonine**	0.07	0.20
**L-tryptophan**	0.01	0.05
**L-valine**	—	0.11
**L-phenylalanine**	—	0.05
**L-isoleucine**	—	0.13
**Dicalcium phosphate**	0.65	0.65
**Limestone**	0.85	1.00
**Salt**	0.30	0.30
**Premix** ^†^	1.00	1.00
***Analyzed nutrient level***		
**Crude protein (%)**	14.06	10.05
***Calculated nutrient level***		
**Net energy (MJ/kg)**	10.38	10.37
**Available phosphorus (%)**	0.25	0.24
**Calcium (%)**	0.52	0.55
**Standardized ileal digestible amino acids (%)** ^‡^		
**Lysine**	0.73	0.73
**Methionine+Cysteine**	0.42	0.42
**Threonine**	0.46	0.46
**Tryptophan**	0.13	0.13
**Valine**	0.54	0.48
**Phenylalanine**	0.59	0.44
**Leucine**	1.06	0.80
**Isoleucine**	0.42	0.39

CP, crude protein.

^**†**^The premix provided per kilogram of diet: 100 mg of zinc as zinc oxide, 100 mg of iron as iron sulphate, 30 mg of manganese as manganous oxide, 0.3 mg of selenium as sodium selenite, 20 mg of copper as copper sulphate, 0.5 mg of iodine as calcium iodate, 1720 μg retinyl acetate, 8.0 mg DL-α-tocopheryl acetate, 25 μg cholecalciferol, 3.0 mg menadione sodium bisulphite, 3.0 mg pyridoxine hydrochloride, 2.0 mg thiamin mononitrate, 6.0 mg riboflavin, 1.0 mg folic acid, 20 μg cyanocobalamin, 30 mg nicotinic acid, 30 mg calcium pantothenate, 300 mg choline.

^**‡**^Values for standardized ileal concentrations of AA were calculated using standardized ileal digestible coefficients provided by Xiong et al., [28]

### Growth Performance

Initial and final body weights and feed consumption were recorded to calculate growth performance including average daily gain (ADG), average daily feed intake (ADFI), and feed conversion ratio (FCR).

### Sample Collection and Carcass Traits

After 41 days of treatments, two pigs per replicate (pen) (40 pigs in total) were randomly selected and transferred to the slaughterhouse. After 12 hours fast, blood samples were collected into 5 mL Eppendorf tubes containing sodium heparin as an anticoagulant (Becton Dickinson Vacutainer Systems, Nanjing, China) via puncture of the anterior vena cava. The blood samples were immediately centrifuged at 3000 × g for 10 min at 4°C to obtain plasma, which were stored a -20°C for further analysis. The 40 pigs were slaughtered by exsanguination after electrical stunning. Within 30 min after slaughter, carcasses were weighed in order to determine the dressing percentage of pigs, the equation was as follows: Dressing percentage = (hot carcass weight / live weight) × 100%. The subcutaneous back fat depth was measured at the first rib, last rib and lumbar as described by Yuan et al., [29]. The average of these three values was used as the back fat thickness. The loin eye area of the *longissimus dorsi* muscle was traced over the 10th rib, and then the area was measured using a Q871 planimeter according to the method described by DeVol et al., [30]. The mathematical model to calculate the lean percentage was as follows: y = 57.742–0.5871 × X_1_ + 0.2023 × X_2_ (X_1_ stands for back fat thickness of last lumbar vertebra, X_2_ represents the distance from the end of gluteus medius to the edge of spinal cord tube) described by Li et al., [31]. Moreover, samples of skeletal muscle (*longissimus dorsi* muscle) were collected and frozen immediately in liquid nitrogen for subsequent analysis.

### Measurement of Plasma Metabolite and Hormone Concentrations

The concentrations of insulin and leptin in the plasma were analyzed according to the commercially available radioimmunoassay kits (Beijing North Institute of Biological Technology, Beijing, China). The concentrations of IGF-1 and SS were performed by using ELISA kits (Nanjing Jiancheng Bioengineering Institute, Nanjing, China). The concentrations of plasma urea nitrogen (PUN) was measured using an urea asssay kit (Nanjing Jiancheng Bioengineering Institute, Nanjing, China).

### Total RNA Isolation and Real-Time PCR Analysis

Total RNA was extracted from skeletal muscle samples using RNAiso Plus reagent (TaKaRa Biotechnology Co. Ltd., Dalian, China). The purity of the total RNA was verified using a NanoDrop 1000 spectrophotometer (Thermo Scientific, Wilmington, DE, USA) at 260 and 280 nm. The OD_260_/OD_280_ ratios of the RNA samples were all between 1.8 and 2.0. Subsequently, the total RNA was treated with DNase I (TaKaRa Biotechnology Co. Ltd., Dalian, China) to remove DNA and reverse transcribed to complementary deoxyribonucleic acid (cDNA) using a PrimeScript RT^TM^ Master Mix kit (TaKaRa Biotechnology Co. Ltd., Dalian, China) according to the manufacturer’s instructions. Real-time PCR was performed using the ABI 7500 Real-Time PCR System (Applied Biosystems, Foster City, CA, USA) with SYBR^®^ Premix Ex Taq^TM^ Kits (Takara Biotechnology Co. Ltd., Dalian, China). The PCR system consisted of 10 μL SYBR Premix Ex Taq, 0.4 μL ROX Reference Dye II, 2 μL cDNA, 6.8 μL double distilled water, and 0.4 μL primer pairs (10 μmol/L forward and 10 μmol/L reverse) in a total volume of 20 μL. The PCR protocols included one cycle at 95°C for 30 s, 40 cycles at 95°C for 5 s and 60°C for 34 s. The PCR products were subjected to a melting curve analysis to verify specific amplifications. β-actin was used as the housekeeping gene to normalize the expression of target genes according to the 2^−∆∆Ct^ method described by Livak and Schmittgen [32]. All samples were measured in triplicate. The primers sequences for target genes are presented in **Table 2.**

**Table 2:** Primer pairs used in the real-time PCR^†^.

Gene	Primer sequence (5' to 3')	Product size(bp)	GenBank accession no.
***Akt***	Forward: CCTGAAGAAGGAGGTCATCG Reversed: TCGTGGGTCTGGAAGGAGTA	123	NM_001159776
***IGF-1***	Forward: GCACATCACATCCTCTTCGC Reversed: ACCCTGTGGGCTTGTTGAAA	165	NM_214256
***IGF-1R***	Forward: ATGGAGGAAGTGACAGGGACTA Reversed: GTGGTGGTGGAGGTGAAGTG	116	XM_003361272
***IR***	Forward: CATACCTGAACGCCAAGAAGTT Reversed: GTCATTCCAAAGTCTCCGATTT	100	XM_003123154
***IRS-1***	Forward: CCCTACTATTTCCCACCAGAAG Reversed: CATTTCCAGACCCTCCTCAG	175	NM_001244489
***FOXO1***	Forward: CGGCATCATCTTCATCGTC Reversed: CTGTCCTCCCACTCCAGGTA	125	NM_214014.2
***FOXO4***	Forward: CTGTCCTACGCCGACCTCAT Reversed: TTGCTGTCACCCTTATCCTTG	103	XM_003135172.2
***MAFbx***	Forward: CCCTCTCATTCTGTCACCTTG Reversed: ATGTGCTCTCCCACCATAGC	104	NM_001044588
***MuRF1***	Forward: GCTGGATTGGAAGAAGATGTAT Reversed: AGGAAAGAATGTGGCAGTGTCT	144	NM_001184756
***β-actin***	Forward: ATGCTTCTAGACGGACTGCG Reversed: GTTTCAGGAGGCTGGCATGA	130	XM_003357928

^**†**^The primer pairs above were reported by Zhang et al., [17].

*Akt*, protein kinase B, also named PKB; *IGF-1*, insulin-like growth factor 1; *IGF-1R*, *IGF-1* receptor; *IR*, insulin receptor; *IRS-1*, insulin receptor substrate 1; *FOXO*, Forkhead Box O; *MAFbx*, muscle atrophy F-box; *MuRF1*, muscle Ring finger 1.

### Western Blot Analysis of mTOR-pathway Proteins

Three pigs per treatment with one pig chosen from one pen were selected for western blot analysis. Specific primary antibodies against total mTOR, phosphorylated mTOR (Ser2448), total 4E-BP1, phosphorylated 4E-BP1 (Thr70), total S6K1, phosphorylated S6K1 (Thr389), and β-actin were purchased from Cell Signaling Technology. Frozen samples of skeletal muscle were homogenized and centrifuged to collect supernatants. Protein concentration in the supernatant fluid was determined using a bicinchonininc acid (BCA) protein assay kit (Sangon Biotec., Shanghai, China). All samples were adjusted to an equal protein concentration and separated through electrophoresis on a 6% (mTOR), 15% (4E-BP1), and 10% (S6K1) polyacrylamide gel. After blocking, proteins were electrophoretically transferred to polyvinylidene difluoride (PVDF) membranes which were incubated with primary antibodies (1:1000) and a secondary antibody (horseradish peroxidase-conjugated goat anti-rabbit IgG, Cell Signaling; 1:3000 dilution in 1% milk). The membranes were developed using a Super Signal West Pico Chemiluminescent Substrate (Thermo Scientific) according to the manufacturer’s instructions, and exposed to a Kodak film. Western blot results were quantified by measuring the band intensities using Scion Image software (Scion Corp., Frederick, MD, USA).

### Statistical Analysis

All experimental data were analyzed using the General Linear Model (GLM) procedures of SPSS (version 16.0, SPSS Inc., Chicago, USA) for a 2 × 2 factorial arrangement of treatments. The statistical model consisted of the effects of protein levels and CS supplemental levels and their interactions. The results were expressed as mean ± SEM. When the interaction was significant, the significance between the treatment differences was identified separately by the least significant difference (LSD) test. The *P*<0.05 was considered significant. Growth performance was analysed with pen as the experimental unit (n = 5); Carcass traits, plasma metabolite and hormone concentrations, and mRNA abundance were analysed with pen as the experimental unit [n = 5 (The means of two pigs per pen were used to represent the pens)]. The protein levels were also analysed with pen as the experimental unit (n = 3).

## Results

### Growth Performance and Carcass Traits

As shown in **Table 3**, dietary protein levels did not affect growth performance and carcass traits (*P*>0.05). However, CS supplementation increased ADG (*P*<0.001) and lean percentage (*P*<0.05), and decreased FCR (*P*<0.05) and back fat (*P*<0.05), while no effects were observed on ADFI, dressing percentage, and loin eye area (*P*>0.05). There were no interactions between dietary protein levels and CS supplementation for all variables (*P*>0.05).

**Table 3:** Effects of dietary crude protein levels and cysteamine (CS) supplementation on growth performance and carcass traits of finishing pigs.

Item	Normal protein		Low protein		*P* value		
	Control	Cysteamine	Control	Cysteamine	P	CS	P × CS
**Initial BW (kg)**	65.87±1.66	65.83±1.21	65.81±1.49	65.87±0.75	0.992	0.992	0.972
**Final BW (kg)**	94.45±1.88	96.72±1.37	93.98±1.74	96.13±1.01	0.736	0.171	0.971
**ADG (g/d)**	697.17±13.88	753.26±8.14	687.15±10.26	738.15±7.14	0.236	<0.001	0.806
**ADFI (kg/d)**	2.53±0.04	2.54±0.12	2.55±0.08	2.46±0.14	0.767	0.731	0.614
**FCR (kg/kg)**	3.63±0.07	3.37±0.15	3.71±0.09	3.33±0.18	0.896	0.028	0.634
**Dressing percentage (%)**	68.56±0.53	68.44±0.29	69.60±1.31	69.73±0.53	0.149	0.993	0.873
**Back fat (cm)**	2.60±0.07	2.29±0.14	2.68±0.10	2.39±0.10	0.426	0.013	0.904
**Loin eye area (cm** ^**2**^ **)**	43.90±0.91	46.10±1.50	42.20±2.56	44.43±1.29	0.331	0.206	0.993
**Lean percentage (%)**	59.24±1.39	62.32±1.24	57.98±0.61	59.58±0.65	0.071	0.038	0.484

BW, body weight; ADG, average daily weight gain; ADFI, average daily feed intake; FCR = feed conversion ratio. Values are means ± SEM. Values within a row with different letters differ (*P*<0.05). All traits in this table were analysed with pen as the experimental unit (n = 5); P, main effect of dietary protein levels; CS, main effect of cysteamine supplementation; P×CS, interaction between main effects of dietary protein levels and cysteamine supplementation.

### Concentrations of Plasma Metabolite and Hormone

As presented in **Table 4**, dietary protein levels did not affect the concentrations of plasma IGF-1, insulin, and leptin (*P*>0.05), while pigs receiving the LP diets had lower concentrations of SS (*P*<0.01) and PUN (*P*<0.001) than those fed the NP diets. However, CS supplementation increased the concentrations of IGF-1 and decreased the concentrations of leptin, SS, and PUN (*P*<0.001), while no effect was observed on the concentrations of insulin (*P*>0.05). There were no interactions between dietary protein levels and CS supplementation for all variables (*P*>0.05).

**Table 4:** Effects of dietary crude protein levels and cysteamine (CS) supplementation on plasma metabolite and hormone concentrations of finishing pigs.

Item	Normal protein		Low protein		*P* value		
	Control	Cysteamine	Control	Cysteamine	P	CS	P × CS
**Insulin (μIU/mL)**	12.22±0.52	13.61±0.82	13.52±0.42	12.94±0.35	0.581	0.475	0.097
**IGF-1 (ng/mL)**	56.79±2.39	72.62±1.01	53.37±1.13	71.42±0.90	0.139	<0.001	0.465
**SS (pg/mL)**	659.25±17.17	500.72±25.71	572.47±17.61	450.43±16.38	0.003	<0.001	0.365
**Leptin (ng/mL)**	2.79±0.02	2.55±0.06	2.79±0.05	2.60±0.02	0.571	<0.001	0.637
**PUN (mmol/L)**	5.65±0.25	4.61±0.15	4.44±0.14	3.77±0.07	<0.001	<0.001	0.283

IGF-1, insulin-like growth factor 1; SS, somatostatin; PUN, plasma urea nitrogen. Values are means ± SEM. Values within a row with different letters differ (*P*<0.05). All traits in this table were analysed with pen as the experimental unit (n = 5). P, main effect of dietary protein levels; CS, main effect of cysteamine supplementation; P×CS, interaction between main effects of dietary protein levels and cysteamine supplementation.

### mRNA Abundance of IGF-1 (Insulin)/Akt1/FOXO Signaling and Their Target Genes in Skeletal Muscle

As shown in **Table 5**, dietary protein levels did not affect the mRNA abundance of studied genes (*P*>0.05). However, CS supplementation increased the mRNA abundance of IGF-1, IGF-1R IRS-1 (insulin receptor substrate 1), and Akt1 (*P*<0.001) and decreased the mRNA abundance of FOXO4 (*P*<0.01) and MAFbx (*P*<0.001) in skeletal muscle, while no effect was observed on the mRNA abundance of IR (insulin receptor), FOXO1, and MuRF1 (*P*>0.05). There were no interactions between dietary protein levels and CS supplementation for the mRNA abundance of all genes (*P*>0.05).

**Table 5:** Effects of dietary crude protein levels and cysteamine (CS) supplementation on mRNA abundance of IGF-1 (insulin)/Akt/FOXO signaling and their target genes in skeletal muscle of finishing pigs.

Item	Normal protein		Low protein		*P* value		
	Control	Cysteamine	Control	Cysteamine	P	CS	P × CS
**IGF-1**	1.00±0.04	1.53±0.08	1.04±0.04	1.64±0.07	0.228	<0.001	0.551
**IGF-1R**	1.00±0.04	1.56±0.04	1.05±0.03	1.50±0.06	0.786	<0.001	0.215
**IR**	1.03±0.04	0.98±0.04	1.06±0.03	0.99±0.05	0.743	0.159	0.851
**IRS-1**	1.01±0.05	1.36±0.02	1.06±0.04	1.43±0.05	0.144	<0.001	0.785
**Akt1**	1.03±0.06	1.62±0.04	1.04±0.03	1.58±0.05	0.756	<0.001	0.577
**FOXO1**	1.02±0.07	0.92±0.06	0.98±0.04	0.91±0.08	0.637	0.197	0.777
**FOXO4**	1.02±0.07	0.83±0.04	1.01±0.07	0.85±0.06	0.891	0.007	0.838
**MAFbx**	1.02±0.05	0.54±0.06	1.05±0.07	0.64±0.03	0.279	<0.001	0.525
**MuRF1**	1.01±0.04	0.93±0.04	1.04±0.06	0.95±0.06	0.562	0.087	0.934

IGF-1, insulin-like growth factor 1; IGF-1R, IGF-1 receptor; IR, insulin receptor; IRS-1, insulin receptor substrate 1. Akt1, protein kinase B, also named PKB; FOXO, Forkhead Box O; MAFbx, muscle atrophy F-box; MuRF1, muscle Ring finger 1. Values are means ± SEM. Values within a row with different letters differ (*P*<0.05). All traits in this table were analysed with pen as the experimental unit (n = 5). P, main effect of dietary protein levels; CS, main effect of cysteamine supplementation; P×CS, interaction between main effects of dietary protein levels and cysteamine supplementation.

### mTOR Pathway Proteins in Skeletal Muscle

**Fig 1:**
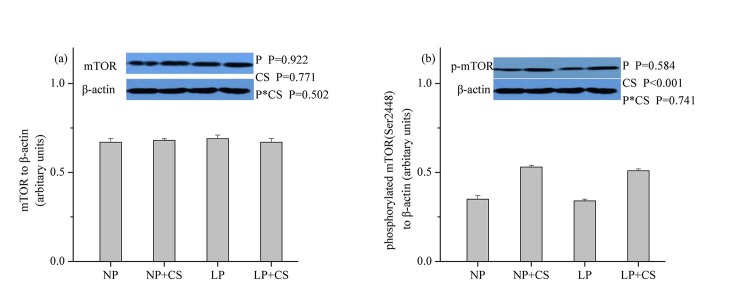
Protein levels of total (a) and phosphorylated (b) mammalian target of rapamycin in skeletal muscle. β-actin was used to normalize the abundance of total and phosphorylated mTOR. Values are means ± SEM. *P<0*.*05* was considered significant difference. Protein levels were analysed with pen as the experimental unit (n = 3). NP, normal protein diet (14% CP); NP+CS, normal protein diet supplemented with cysteamine; LP, low-protein diet (10% CP); LP+CS, low-protein diet supplemented with cysteamine. P, main effect of dietary protein levels; CS, main effect of cysteamine supplementation; P×CS, interaction between main effects of dietary protein levels and cysteamine supplementation.

**Fig 2:**
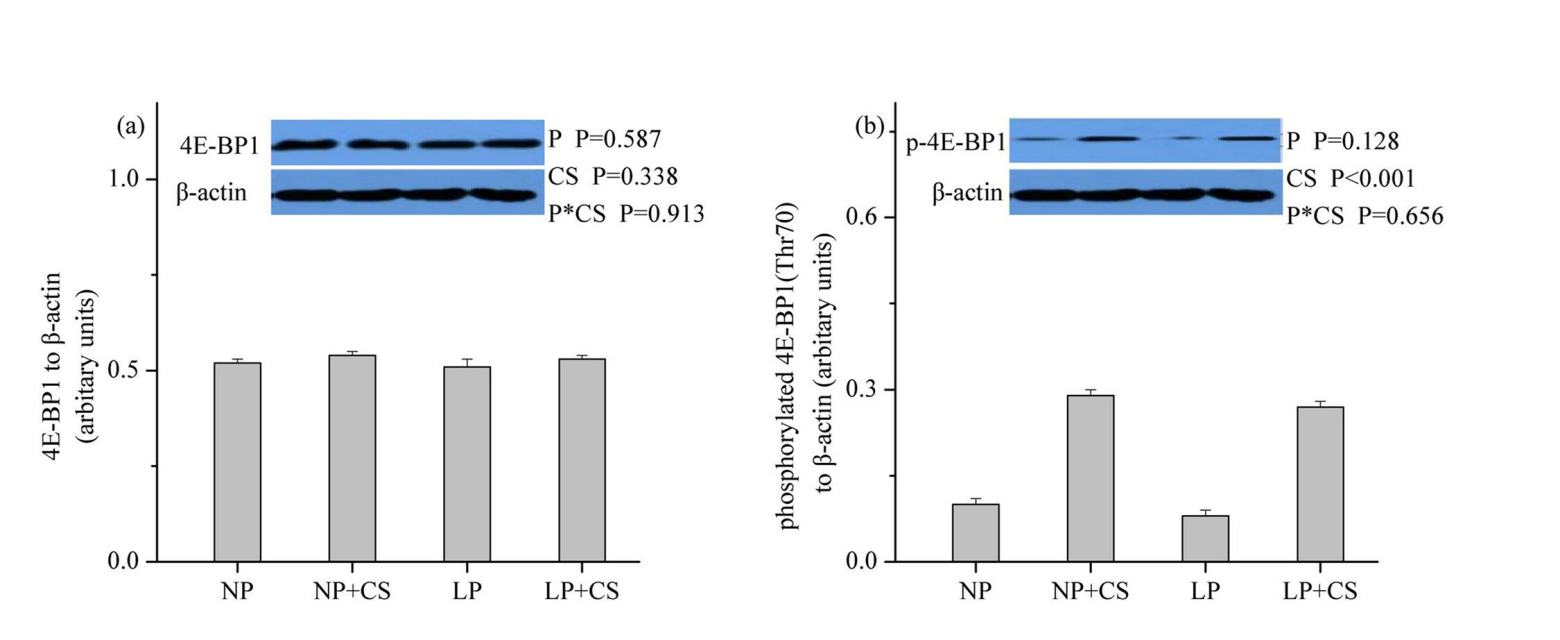
Protein levels of total (a) and phosphorylated (b) eIF4E-binding protein 1 in skeletal muscle. β-actin was used to normalize the abundance of total and phosphorylated 4E-BP1. Values are means ± SEM. *P<0*.*05* was considered significant difference. Protein levels were analysed with pen as the experimental unit (n = 3). NP, normal protein diet (14% CP); NP+CS, normal protein diet supplemented with cysteamine; LP, low-protein diet (10% CP); LP+CS, low-protein diet supplemented with cysteamine. P, main effect of dietary protein levels; CS, main effect of cysteamine supplementation; P×CS, interaction between main effects of dietary protein levels and cysteamine supplementation.

**Fig 3:**
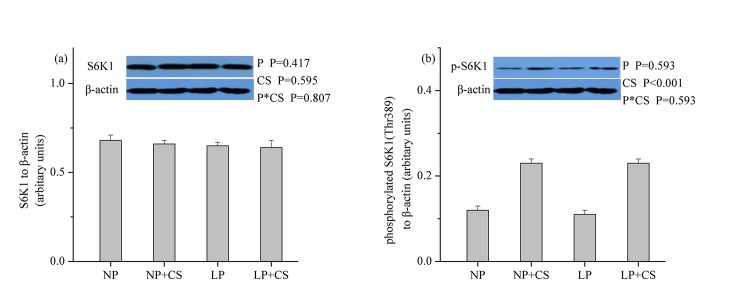
Protein levels of total (a) and phosphorylated (b) p70S6K1 in skeletal muscle. β-actin was used to normalize the abundance of total and phosphorylated S6K1. Values are means ± SEM. *P<0*.*05* was considered significant difference. Protein levels were analysed with pen as the experimental unit (n = 3). NP, normal protein diet (14% CP); NP+CS, normal protein diet supplemented with cysteamine; LP, low-protein diet (10% CP); LP+CS, low-protein diet supplemented with cysteamine. P, main effect of dietary protein levels; CS, main effect of cysteamine supplementation; P×CS, interaction between main effects of dietary protein levels and cysteamine supplementation.

As shown in **Figs 1–3**, dietary protein levels did not affect the protein levels for total and phosphorylated mTOR, 4E-BP1, or S6K1 (*P*>0.05). However, CS supplementation increased the protein abundance of phosphorylated mTOR, 4E-BP1, and S6K1 (*P*<0.001), while no effect was observed on the protein abundance of total mTOR, 4E-BP1, or S6K1 (*P*>0.05). There were no interactions between dietary protein levels and CS supplementation for the protein levels of total and phosphorylated mTOR, 4E-BP1, or S6K1 (*P*>0.05).

## Discussion

Growth performance and carcass characteristics vary when dietary CP is reduced by approximately 4 percentage units, although the LP diets are often supplemented with EAA to meet the NRC AA requirements of pigs. Kendall [33] reported that when grower pigs fed a diet that was reduced in CP by approximately 4 percentage units and lysine, threonine, tryptophan, and methionine were supplemented, the growth performance was decreased, results that were similar to those reported by Gómez et al., [34]. In contrast, Shriver et al., [35] demonstrated that when dietary CP concentrations were reduced by 4 percentage units and lysine, threonine, tryptophan, methionine isoleucine, and valine were supplemented, the growth performance of finishing pigs was maintained, but a higher back fat thickness was observed in the pigs fed LP diets, which was consistent with the work by Kerr et al., [36]. In the present study, enough EAA were added to the LP diets to meet the NRC AA requirements of pigs and maintain the balanced supply of eight EAA, and no differences in the growth performance and carcass traits were found in the LP diets. The reason for this discrepancy may be ascribed to a better balance of EAA by simultaneously supplementing isoleucine, valine and phenylalanine to LP diets apart from lysine, methionine, threonine and tryptophan to meet animals’ requirements in our study [37]. In addition, experimental evidence from studies with animals have demonstrated that crystalline AA has high-nutritional values when they are added to a diet deficient in those AA, thereby ensuring the maximum utilization of dietary protein to maintain the growth performance of pigs [38]. Previous studies have revealed that LP diets may have higher NE values. In that case, pigs expend less metabolic energy in the process of deamination of excess AA and body protein turnover, which in turn reduces heat production. Then the extra energy may be deposited as adipose tissue. The diets used in our experiment were formulated to be isoenergetic on a NE basis instead of a metabolizable energy (ME) basis [36,39]. Thus feeding LP diets did not affect carcass characteristics.

In the present study, CS supplementation did not affect ADFI, but significantly increased ADG and decreased FCR, which was in agreement with the finding of Yang et al., [4], who concluded that dietary CS supplementation (30 mg/kg) caused significant increases in the growth rate and feed conversion efficiency of finishing pigs, but no difference was observed in the feed intake. It is well known that CS supplementation could increase the secretion of GH and IGF-1 **(Table 4)**. IGF-1 is mainly synthesized in the liver and functions as an endocrine hormone through blood circulation. Additionally, most extrahepatic tissues (such as muscle) can secrete IGF-1, which can function as an autocrine or paracrine growth stimulator [22, 40]. Furthermore, some studies have demonstrated that IGF-1 not only can induce skeletal muscle hypertrophy by activating the IGF-1/Akt/mTOR pathway but also can inhibit protein degradation by suppressing the expression of MAFbx and MuRF1 mRNAs [12]. Therefore, we speculate that the increased growth performance may be associated with the alterations of protein synthesis and breakdown signaling pathways by increasing IGF-1 levels. We also observed a lower back fat and a higher lean percentage in CS supplementation treatments, which was consistent with the work on CS by Yang et al., [4]. This fat reduction may be likely attributed to the direct action of GH [3,41].

Dietary protein restriction has been reported to decrease plasma concentrations of insulin and IGF-1 [7,42]. However, plasma IGF-1 and insulin concentrations were not affected by LP diets in our study. These results suggested that these hormone concentrations were not positively correlated with the dietary protein levels, if we maintained the balanced supply of EAA in the diets to meet the animals’ nutrient requirements. The lack of an effect of LP diets on insulin may be because the diets were formulated to contain constant levels of NE. Growth in pigs is regulated primarily by the brain neuroendocrine GH-IGF axis, and SS is the major inhibitor of GH secretion in swine [4]. CS functions as a specific inhibitor of SS to increase GH secretion, causing an increase in IGF-1 concentrations, which in turn promotes protein deposition [3,5]. In our study, CS supplementation resulted in a marked increase in plasma IGF-1 levels and a decrease in SS concentrations, despite the lack of change in insulin levels, indicating that the signaling pathway leading to protein metabolism was mainly regulated by IGF-1, rather than insulin following CS supplementation. Interestingly, a significant decrease in SS was observed in pigs fed LP diets. This may be explained by the feedback regulation mechanisms in these animals.

Leptin, which is synthesized in fat cells, has been implicated in the regulation of feed intake and energy metabolism. Plasma leptin concentrations are positively correlated with carcass backfat thickness [7,43]. In our present study, CS supplementation decreased plasma leptin levels, concurrent with the changes in back fat. However, feed intake was not regulated by leptin. Possibly because the changes in the leptin concentrations were not sufficient to affect feed intake.

Consistent with previous studies [8,44], PUN was reduced as the level of CP was decreased in the present study. Urea is generally synthesized when AA intake surpasses the amount needed by the organism for the synthesis of proteins [45]. The reduction in PUN in pigs fed a LP diet is indicative of a better AA balance and a reduction in excess AA in the diet, which may depend on the reduced AA catabolism and greater synthesis of protein [35]. Moreover, PUN was also decreased by CS supplementation, indicating more efficient utilization of N. The possible mechanisms could be summarized as: CS may stimulate protein deposition via the IGF1/Akt/mTOR pathway, thus more AA would be involved in the synthesis of proteins, and deamination and transamination processes would be blocked, leading to lower PUN concentrations [21].

The biological functions of IGF-1 and insulin are mediated mainly through the tyrosine kinases receptors, IGF-1R and IR. The binding of IGF-1 and insulin to their receptors can recruit IRS1 adaptor proteins, leading to the activation of the phosphatidylinositol-3-kinase (PI3K)/Akt/mTOR signaling pathway, which regulates cell growth and proliferation [18,46]. In the current study, CS supplementation increased the mRNA abundance of IGF-1, IGF-1R, and IRS-1, but did not affect the mRNA abundance of IR. Similar results were obtained by Liu et al., [22], who reported upregulation of IGF-1 and IGF-1R mRNA levels, but not IR mRNA level in the muscle following supplementation with 70 mg/kg CS. Tse et al., [2] also reported that chronic CS treatment could elevate IGF-1 mRNA level in the muscle of fish. Furthermore, our data showed that the results in our study were parallel to the changes in plasma IGF-1 and insulin concentrations, indicating that insulin secretion and IR gene expression were less sensitive to changes in CS supplementation. Thus, the higher mRNA levels of IGF-1, IGF-1R, and IRS-1 in muscle supported the hypothesis that CS may affect the growth rate of muscle growth during development through IGF-1 signaling. Additionally, few studies have reported the effects of dietary protein levels on mRNA expression of components of the insulin (IGF-1) signaling pathway. In the present study, there were no differences in mRNA abundance of IR, IRS-1, IGF-1, and IGF-1R between pigs fed NP diets and pigs fed LP diets, which was consistent with the work by Brameld et al., [47], who showed that dietary protein levels did not affect skeletal muscle IGF-1 expression. The changes in mRNA expression may be associated with the changes in plasma IGF-1 and insulin concentrations.

mTOR plays a crucial role in the control of protein synthesis through its two downstream targets S6K1 and 4EBP1 and is regulated by hormonal factors and AA [13,26]. In this study, we found that mTOR signaling was not affected by differences in dietary protein levels, which was in agreement with the finding of Deng et al., [26], who concluded that the protein abundance of phosphorylated mTOR, 4E-BP1, and S6K1 was similar in skeletal muscle of pigs fed diets containing 16.7% and 20.7% CP. Thus, our findings indicated that LP diets supplemented with EAA could facilitate maintenance of protein synthesis signaling pathway. In contrast, CS supplementation increased the protein abundance of phosphorylated mTOR, 4E-BP1 and S6K1, which was likely due to the rise in circulating levels of IGF-1. In support of this view, Han et al., [48] reported that IGF-1 could stimulate proliferation and protein synthesis in myogenic satellite cells via activation of Akt/mTOR signaling pathway *in vitro*.

Akt, an important signaling protein, is not only capable of activating protein synthesis pathways but also simultaneously inhibits protein degradation pathways by phosphorylating and inactivating FOXO transcription factors [18]. Phosphorylation and inactivation of FOXO results in exportation of FOXO from the nucleus to the cytoplasm, causing downregulation of MAFbx and MuRF1, two key regulators in the UPP pathway, this then prevents protein degradation [17]. In our present study, no differences in mRNA abundance of Akt1, FOXO4, FOXO1, MAFbx, and MuRF1 were found between pigs fed NP diets and pigs fed LP diets, indicating that the changes in dietary protein concentration did not affect the UPP pathway. To date, few studies have examined the effects of dietary protein levels on the Akt/FOXO/UPP pathway. Sandri et al., [49] reported that the balance between protein synthesis and protein degradation was determined by IGF-1 and insulin levels. Hence, it is reasonable to speculate that the lack of change in the mRNA expression of Akt/FOXO signaling factors could be attributed to the lack of change in plasma IGF-1 and insulin concentrations. MAFbx and MuRF1 are also modulated via mechanisms involving the Akt/mTOR pathway by AA availability *in vivo*. The balanced AA composition in diet could contribute to the balanced AA concentrations in the plasma, which could regulate the mTOR pathway directly [23]. Additionally, CS supplementation increased the mRNA abundance of Akt1 and decreased the mRNA abundance of FOXO4 and MAFbx. Consistent with these findings, the protein turnover trial showed a decrease in protein breakdown following CS supplementation [3]. Interestingly, *in vitro* and *in vivo* studies have shown that IGF-1 stimulates muscle hypertrophy by suppressing protein degradation and the expression of atrophy-induced ubiquitin ligases, MAFbx and MuRF1 [5,18]. Thus it is possible that the beneficial effects of CS supplementation on muscle protein degradation can be explained by the higher plasma IGF-1 concentrations. Moreover, the lack of change in MuRF1 compared with the significant decrease in MAFbx indicated that MAFbx mRNA was much more sensitive to the changes in IGF-1 than MuRF1 mRNA, similar results were also obtained by Sacheck et al., [5]. In addition, we observed no difference in the mRNA expression of FOXO1, suggesting that FOXO4 was more sensitive to CS supplementation. Such a difference may stem from variations in tissue-specific gene expression; for example, FOXO1 is highly expressed in adipose tissue, whereas FOXO4 is highly expressed in muscle [20].

In conclusion, there were no interactions between dietary protein levels and CS supplementation in terms of growth performance and other traits in pigs. Dietary protein levels and CS supplementation influenced growth and protein metabolism through independent mechanisms in pigs. In addition, LP diets supplemented with enough EAA to meet the NRC AA requirements of pigs and maintain the balanced supply of eight EAA did not affect growth performance and other traits except the concentrations of SS and PUN, which may be attributed to the maintenance of protein synthesis and degradation signaling. Moreover, CS supplementation improved growth performance, possibly by mediating plasma IGF-1 concentrations, thereby promoting mTOR signaling and suppressing Akt/FOXO signaling in skeletal muscle of finishing pigs.

## Supporting Information

S1 Table The minimal data for growth performance and carcass traits of finishing pigs. (XLSX)

S2 Table The minimal data for plasma metabolite and hormone concentrations of finishing pigs. (XLSX)

S3 Table The minimal data for mRNA abundance of IGF-1 (insulin)/Akt/FOXO signaling and their target genes in skeletal muscle of finishing pigs. (XLSX)

S4 Table The minimal data for protein levels of total and phosphorylated mammalian target of rapamycin in skeletal muscle. (XLSX)

S5 Table The minimal data for protein levels of total and phosphorylated eIF4E-binding protein 1 in skeletal muscle. (XLSX)

S6 Table The minimal data for protein levels of total and phosphorylated p70S6K1 in skeletal muscle. (XLSX)
